# HBSP: a hybrid bilinear and semidefinite programming approach for aligning partially overlapping point clouds

**DOI:** 10.1038/s41598-024-79744-x

**Published:** 2024-12-03

**Authors:** Wei Lian, Fei Ma, Zhesen Cui, Hang Pan

**Affiliations:** Department of Computer Science, Changzhi University, Changzhi, 046011 Shanxi China

**Keywords:** Branch-and-bound, Semidefinite programming, Point cloud registration, Linear assignment, Bilinear relaxation, Computational science, Computer science

## Abstract

In many applications, there is a need for algorithms that can align partially overlapping point clouds while remaining invariant to corresponding transformations. This research presents a method that achieves these goals by minimizing a binary linear assignment-least squares (BLALS) energy function. First, we reformulate the BLALS problem as the minimization of a quadratic function with quadratic and linear constraints through variable substitution. By utilizing semidefinite relaxation and the convex envelope of bilinear monomials, we relax the problem to create a lower bound that can be solved using linear assignment and low-dimensional semidefinite programming. Additionally, we develop a branch-and-bound (BnB) algorithm that only branches over the transformation variable, which enhances convergence. Experimental results show that, compared to state-of-the-art approaches, the proposed method is robust against non-rigid deformation and outliers when the outliers are separate from the inliers. However, its robustness decreases when outliers are mixed with inliers. The run time of our method is relatively high due to the need to solve a semidefinite program in each iteration of the BnB algorithm.

## Introduction

Point cloud registration (also known as scan matching or point cloud alignment) involves finding a spatial transformation (like scaling, rotation, and translation) to align two point clouds. This task is crucial in fields like computer vision, robotics, and remote sensing. However, it presents several challenges, including non-rigid deformations, positional noise, partial overlap, and lack of initial coarse alignment. To tackle these issues, various approaches have been proposed, which can be broadly divided into heuristic-based methods and global methods.

1. *Heuristic-based methods*
  (i) *Correspondence-free methods* The Coherent Point Drift (CPD) method^1^ frames point matching as fitting a Gaussian mixture model (GMM) from one point cloud to another. Similarly, the Gaussian Mixture Model Registration (GMMREG) method^2^ uses two GMMs to represent point clouds and minimizes the distance between them. Support Vector Parameterized Gaussian Mixtures (SVGMs)^3^ also utilize sparse Gaussian components to represent point clouds.
    To enhance efficiency, filtering techniques have been introduced to address the correspondence problem^4^. Additionally, to tackle density variation in point clouds, methods have been developed to model the scene’s structure^5^. A hierarchical Gaussian mixture representation has been created to improve both the speed and accuracy of registration^6^.
    While these methods do not explicitly establish point correspondences, making them computationally efficient, they generally perform worse than correspondence-based methods when dealing with non-rigid deformations.
  (ii) *Simultaneous pose and correspondence* The Iterative Closest Point (ICP) method^7,8^ alternates between recovering point correspondences and updating spatial transformations. Although efficient, its reliance on discrete point correspondences makes it prone to local minima.
    In contrast, the Robust Point Matching (RPM) method^9^ formulates registration as minimizing a binary linear assignment-least squares (BLALS) energy function:
    1 $$\begin{aligned} E(\textbf{P}, \varvec{\theta }) = \sum _{i,j} p_{ij} \Vert \textbf{T}(\textbf{x}_i | \varvec{\theta }) -\textbf{y}_j \Vert ^2 \end{aligned}$$
    Here, $\{\textbf{x}_i\}$ and $\{\textbf{y}_j\}$ represent the coordinates of the two point clouds being aligned. The correspondence matrix $\textbf{P}$ contains elements $p_{ij} \in \{0,1\}$, indicating whether point $\textbf{x}_i$ matches point $\textbf{y}_j$. The function $\textbf{T}(\cdot | \varvec{\theta })$ denotes a spatial transformation parameterized by $\varvec{\theta }.$
    RPM relaxes point correspondences by allowing fuzzy values and uses deterministic annealing for gradual refinement. Sofka et al. enhanced this approach by employing the covariance matrix of transformation parameters to determine the fuzziness of point correspondences, which improves robustness against missing or extraneous structures^10^.
    Our method shares similarities with these approaches, as it models both spatial transformations and correspondences. However, we adopt a different strategy by globally minimizing our objective function, enhancing robustness to disturbances.
  (iii) *Limitations* The heuristic-based methods discussed above are generally not rotation-invariant and may fail without good initial coarse alignment. They can also struggle when the registration problem becomes particularly challenging.
2. *Global methods*
  (i) *Correspondence-free methods* The Branch-and-Bound (BnB) algorithm is a well-known global optimization technique. It is used to align 3D shapes based on Lipschitz theory in^11^. However, this method does not account for outliers or occlusions. In another application, BnB is used to recover 3D rigid transformation^12^, but it requires known correspondences in advance, which limits its applicability.
    The Globally Optimal ICP (Go-ICP) method^13^ optimizes the objective of ICP globally. The globally-optimal Gaussian mixture alignment (GOGMA) method^14^ registers point clouds by aligning GMMs constructed from the original point clouds. The Fast Rotation Search (FRS) method uses stereographic projection to efficiently recover 3D rotation between two point clouds^15^. Additionally, a rotation-invariant feature proposed in^16^ leads to an efficient BnB-based registration algorithm. A Bayesian nonparametric mixture and a novel tessellation approach for rotation space are introduced in^17^, resulting in an effective alignment algorithm.
    These methods focus solely on rigid transformations and do not explicitly establish point correspondences, making them less robust to non-rigid deformations. In contrast, our BnB-based method can handle affine transformations and model point correspondences, allowing it to better manage non-rigid deformations.
  (ii) *Simultaneous pose and correspondence* The Fast Global Registration (FGR) method^18^ optimizes a robust objective by first transforming it into a form similar to (1), leveraging the duality between line processes and robust estimation. TEASER++^19^ uses a graph-theoretic approach to decouple rotation, scale, and translation estimations, employing truncated least squares for each subproblem.

Frank Pfeuffer et al. use the BnB technique to optimize (1) by branching on either the correspondence or transformation variables^20^. However, this method does not scale well with the problem size, primarily due to the high dimensionality of the search space and an inefficient bounding scheme.

Recent approaches have focused on utilizing the low-rank structure of the BLALS objective. By eliminating the spatial transformation variable, methods such as^21^ and^22^ simplify (1) into concave functions of the point correspondence variable. This reveals low-rank structures, enabling efficient global optimization using BnB techniques.

However, the method from^21^ is only applicable when one point cloud can be embedded within another, while method^22^ lacks invariance to corresponding transformations. Although methods^23^ and^24^ have addressed the transformation invariance issue, they still struggle with high-dimensional branching spaces, resulting in slow convergence.

All these methods^21–24^ share a common strategy: they eliminate spatial transformations from the objective (1) to create functions based solely on point correspondences. However, the resulting functions tend to be complex, leading to challenges such as high-dimensional branching spaces and intricate optimization strategies.

In contrast, an alternative approach by^25^ suggests retaining spatial transformations. Based on the observation that (1) is a cubic polynomial, this method employs trilinear and bilinear relaxation to derive a lower bound function, offering a different pathway for addressing optimization challenges.

## Contributions

Building on the method introduced in^25^, we propose a new hybrid bilinear and semidefinite programming (HBSP) approach based on a BnB strategy to optimize the BLALS objective (1). Our algorithm offers several advantages:

(i) The algorithm in^25^ is complex due to the need to reformulate function (1) to ensure that one of the variables in the trilinear monomial includes the origin within its range. In contrast, our method employs bilinear relaxation to establish the lower bound function, eliminating this requirement. As a result, our algorithm is more straightforward.
(ii) The method^25^ develops separate algorithms for 2D and 3D cases, making their approach seem complicated. In contrast, we develop a unified algorithm that works for both dimensions. This makes our solution cleaner and more streamlined.
(iii) The optimization of the lower bound function can be divided into two distinct tasks: linear assignment and low-dimensional semidefinite programming. Both tasks can be solved with guarantees of global optimality.
(iv) Our approach retains the beneficial features identified in^25^, such as the ability to handle partial overlap, invariance to corresponding transformations, and the use of a low-dimensional branching space.

## Bilinear convex envelope linearization

The well-known convex envelope of a bilinear monomial $xy$ under the constraints ${\underline{x}} \le x \le {\overline{x}}$ and ${\underline{y}} \le y \le {\overline{y}}$ can be described as^26^:

2 $$\begin{aligned} {\left\{ \begin{array}{ll} (xy)_{l1} \triangleq {\underline{x}}y + x{\underline{y}} - {\underline{x}}{\underline{y}} \le xy ,\\ (xy)_{l2} \triangleq {\overline{x}}y + x{\overline{y}} - {\overline{x}}{\overline{y}} \le xy \end{array}\right. } \end{aligned}$$

There are two ways to use this formula when designing the lower bound problem:

(i) Integrate the term $\max \{(xy)_{l1},(xy)_{l2}\}$ into the lower bound objective function. However, this incorporation leads to non-linearity in the resultant function.
(ii) Formulate a subproblem in the format $\min \{\alpha \,|\, \alpha \ge (xy)_{l1}, \alpha \ge (xy)_{l2}\}$ within the lower bound problem. This approach, however, increases the complexity of constraints and raises issues regarding the feasibility of efficient optimization via combinatorial optimization algorithms.

Following the method proposed in^25^, we advocate using the average of the convex envelope components:

3 $$\begin{aligned} (xy)_{l} \triangleq \frac{1}{2} \sum _i (xy)_{li} =\frac{{\underline{x}}+{\overline{x}}}{2} y + \frac{{\underline{y}}+{\overline{y}}}{2} x -\frac{{\underline{x}}{\underline{y}}+{\overline{x}}{\overline{y}}}{2} \le xy \end{aligned}$$

as the lower bound function for $xy.$ This choice has the advantage of making the lower bound function affine, thereby facilitating efficient optimization by combinatorial optimization algorithms.

The method^25^ also shows that for $(xy)_l$ to converge to $xy$, it is sufficient to branch over just one of the variables, $x$ or $y$, that make up the bilinear monomial $xy$. This is a crucial property, and the good convergence of the proposed algorithm hinges on this fact.

## Problem formulation

Consider two point clouds, $\mathscr {X} = \{ \textbf{x}_i \, | \, 1 \le i \le n_x \}$ and $\mathscr {Y} = \{ \textbf{y}_j \, | \, 1 \le j \le n_y \}$, in $\mathbb {R}^{n_d}$. The vectors $\textbf{x}_i$ and $\textbf{y}_j$ represent the coordinates of points $i$ and $j$, respectively.

To ensure the feasibility of our point cloud registration problem, we make the following assumptions:

(i) *Linear transformation* We assume that the transformation $\textbf{T}(\cdot | \varvec{\theta })$ is linear with respect to its parameters $\varvec{\theta }$: $\textbf{T}(\textbf{x}_i | \varvec{\theta }) = \textbf{J}_i \varvec{\theta },$ where $\textbf{J}_i$ is the Jacobian matrix at point $\textbf{x}_i$. This formulation captures various transformations, including 2D similarity/affine and 3D affine transformations.
(ii) *One-to-one matching and known number of matches* We assume that point matching is one-to-one, expressed with the following constraints on ${\textbf{P}}$: $\textbf{P} \textbf{1}_{n_y} \le \textbf{1}_{n_x}, \textbf{1}_{n_x}^\top \textbf{P} \le \textbf{1}_{n_y}^\top ,$ where $\textbf{1}_{n_x}$ is the $n_x$-dimensional vector of all ones. Additionally, the total number of point matches, denoted as $n_p$, is known a priori: $\textbf{1}_{n_x}^\top \textbf{P} \textbf{1}_{n_y} = n_p.$ These assumptions are crucial for efficiently solving the lower bounding problem using linear assignment algorithms.

Given these assumptions, we can formulate the minimization of the objective function (1) under the constraints of $\textbf{P}$ and $\varvec{\theta }$ as follows:

4a $$\begin{aligned} \min _{\textbf{P}, \varvec{\theta }} E(\textbf{P}, \varvec{\theta })&= \sum _{i,j} p_{ij} \Vert \textbf{J}_i \varvec{\theta }-\textbf{y}_j \Vert ^2 \nonumber \\&=\sum _{i,j} p_{ij} \varvec{\theta }^\top \textbf{J}^\top _i \textbf{J}_i \varvec{\theta } -2 \sum _{i,j} p_{ij} \varvec{\theta }^\top \textbf{J}^\top _i {\textbf{y}}_j + \sum _{i,j} p_{ij} \Vert {\textbf{y}}_j\Vert ^2\nonumber \\&= {\varvec{\theta }}^\top {\textbf{J}}^\top (\text {diag}({\textbf{P}} {\textbf{1}}_{n_y}) \otimes {\textbf{I}}_{n_d}) {\textbf{J}} {\varvec{\theta }} -2{\varvec{\theta }}^\top {\textbf{J}}^\top ({\textbf{P}}\otimes {\textbf{I}}_{n_d}) {\textbf{y}} + \textbf{1}_{n_x}^\top {\textbf{P}} \widetilde{{\textbf{y}}} \nonumber \\&= {\varvec{\theta }}^\top \widetilde{{\textbf{J}}}^\top (({\textbf{P}} {\textbf{1}}_{n_y}) \otimes {\textbf{I}}_{n_\theta }) {\varvec{\theta }} -2{\varvec{\theta }}^\top {\textbf{J}}^\top ({\textbf{P}}\otimes \textbf{I}_{n_d}) {\textbf{y}} + {\textbf{1}}_{n_x}^\top {\textbf{P}} \widetilde{{\textbf{y}}} \end{aligned}$$

4b $$\begin{aligned} \text {s.t.} \quad \textbf{P} \textbf{1}_{n_y} \le&\textbf{1}_{n_x} , \quad \textbf{1}_{n_x}^\top \textbf{P} \le \textbf{1}_{n_y}^\top , \quad \textbf{1}_{n_x}^\top \textbf{P} \textbf{1}_{n_y} = n_p, \quad \textbf{P} \ge 0, \quad \underline{\varvec{\theta }} \le \varvec{\theta } \le \overline{\varvec{\theta }} \end{aligned}$$

Here, $n_\theta$ denotes the dimensionality of ${\varvec{\theta }}$, $\otimes$ indicates the Kronecker product, and ${\textbf{I}}_{n_d}$ represents the $n_d\times n_d$ identity matrix. The vectors $\textbf{y}$ and $\widetilde{\textbf{y}}$ are defined as: $${\textbf{y}}\triangleq \begin{bmatrix} {\textbf{y}}_1^\top , \dots , {\textbf{y}}_{n_y}^\top \end{bmatrix}^\top$$, $$\widetilde{\textbf{y}}\triangleq \begin{bmatrix} \Vert \textbf{y}_1\Vert _2^2, \dots , \Vert {\textbf{y}}_{n_y}\Vert _2^2 \end{bmatrix}^\top$$. The matrices ${\textbf{J}}$ and $\widetilde{\textbf{J}}$ are defined as: $${\textbf{J}} \triangleq \begin{bmatrix} {\textbf{J}}^\top _1, \dots , {\textbf{J}}^\top _{n_x} \end{bmatrix}^\top$$, $$\widetilde{\textbf{J}}\triangleq \begin{bmatrix} \textbf{J}_1^\top \textbf{J}_1, \ldots , \textbf{J}_{n_x}^\top \textbf{J}_{n_x} \end{bmatrix}^\top$$. The vectors $\underline{\varvec{\theta }}$ and $\overline{\varvec{\theta }}$ represent the lower and upper bounds of $\varvec{\theta }$, respectively.

To facilitate designing an optimization algorithm for *E*, we first need to vectorize the matrix $\textbf{P}$ into a vector $\textbf{p} \triangleq \text {vec}(\textbf{P})$, where, contrary to convention, we define the vectorization operator $\text {vec}(\cdot )$ to concatenate rows instead of columns. Utilizing the formula $\text {vec}({\textbf{C}}_1 {\textbf{C}}_2 \textbf{C}_3) = ({\textbf{C}}_1 \otimes {\textbf{C}}_3) \text {vec}({\textbf{C}}_2)$ for any multipliable matrices ${\textbf{C}}_1$, ${\textbf{C}}_2$, and ${\textbf{C}}_3$, we can express $E$ in terms of the vector $\textbf{p}$ as follows^22^:

5 $$\begin{aligned} E({\textbf{p}},{\varvec{\theta }})=\varvec{\theta }^\top \text {mat} ((\widetilde{\textbf{J}}^\top \otimes {\textbf{I}}_{n_\theta }) \textbf{W}^{n_x,1}_{n_\theta } ({\textbf{I}}_{n_x} \otimes {\textbf{1}}_{n_y}^\top ) {\textbf{p}}) \varvec{\theta }- 2\varvec{\theta }^\top (\textbf{J}^\top \otimes {\textbf{y}}^\top ){\textbf{W}}^{n_x,n_y}_{n_d} {\textbf{p}} + ({\textbf{1}}_{n_x} \otimes \widetilde{{\textbf{y}}} )^\top {\textbf{p}} \end{aligned}$$

In this expression, the operator $\text {mat}(\cdot )$ reconstructs a matrix from a vector, serving as the inverse of the $\text {vec}(\cdot )$ operator. The matrix ${\textbf{W}}^{m,n }_{d}$ is defined as^21^:

$$\begin{aligned} {\textbf{W}}^{m,n}_{d}\triangleq {\textbf{I}}_{m} \otimes \begin{bmatrix} {\textbf{I}}_{n} \otimes ({\textbf{e}}_{d}^1)^\top , \dots , {\textbf{I}}_{n} \otimes ({\textbf{e}}_{d}^{d})^\top \end{bmatrix}^\top \end{aligned}$$

which is an $m n d^2 \times m n$ matrix satisfying $\text {vec}(\textbf{C} \otimes \textbf{I}_{d}) = \textbf{W}^{m,n}_{d} \text {vec}(\textbf{C})$ for any $m\times n$ matrix ${\textbf{C}}$. Here, the $d$-dimensional vector $\textbf{e}_{d}^i$ has a single unity element at coordinate $i$. The matrix ${\textbf{W}}^{m,n}_{d}$ is large but sparse, and it can be implemented using the speye function in MATLAB.

Next, we define the vector $\varvec{\rho } \triangleq \textbf{1}_{n_x} \otimes \widetilde{\textbf{y}}$ and the matrices ${\textbf{A}}\triangleq (\textbf{J}^\top \otimes {\textbf{y}}^\top )\textbf{W}^{n_x,n_y}_{n_d}$ and $\textbf{B}\triangleq (\widetilde{\textbf{J}}^\top \otimes \textbf{I}_{n_\theta }) {\textbf{W}}^{n_x,1}_{n_\theta } ({\textbf{I}}_{n_x} \otimes {\textbf{1}}_{n_y}^\top )$, Thus, we can rewrite $E$ in a more concise form:

6 $$\begin{aligned} E({\textbf{p}},{\varvec{\theta }})=\varvec{\theta }^\top \text {mat} ({\textbf{B}}{\textbf{p}}) \varvec{\theta }- 2\varvec{\theta }^\top {\textbf{A}}{\textbf{p}} + {\varvec{\rho }}^\top {\textbf{p}} \end{aligned}$$

## BnB based optimization

### Lower bound

By introducing a new matrix variable $\varvec{\Theta }=\theta \theta ^\top$, the above optimization problem can be reformulated as minimizing a quadratic function subject to quadratic and linear constraints:

7a $$\begin{aligned} & \min _{ \varvec{\Theta }, \theta ,{\textbf{p}}} E(\varvec{\Theta },\theta ,{\textbf{p}})= \text {trace} ( \text {mat} ( {\textbf{B}}{\textbf{p}} ) \varvec{\Theta } ) {-} 2\theta ^\top {\textbf{A}}{\textbf{p}} {+} \varvec{\rho }^\top {\textbf{p}} \end{aligned}$$

7b $$\begin{aligned} & s.t.\ \varvec{\Theta } = \theta \theta ^\top ,\ {\textbf{p}}\in \Omega , \ \underline{\varvec{\theta }}\le \varvec{\theta }\le \overline{\varvec{\theta }} \end{aligned}$$

where ’trace’ denote the trace of a square matrix and $\Omega$ denotes the feasible region of *p*, as defined in (4b).

This problem is still difficult to optimize due to the existence of bilinear terms $\text {trace} ( \text {mat} ( {\textbf{B}}{\textbf{p}} )\varvec{\Theta })$ and ${-} 2\varvec{\theta }^\top {\textbf{A}}{\textbf{p}}$ in the objective function and quadratic equality constraint $\varvec{\Theta } = \theta \theta ^\top$. To address these issues, for the bilinear terms, we can use formula (3) to relax these bilinear terms into linear terms:89

where based on (3), the constant matrices ${\textbf{H}}^0$, ${\textbf{H}}^1$ and ${\textbf{H}}^2$ and vectors ${\textbf{g}}^0$, ${\textbf{g}}^1$ and ${\textbf{g}}^2$ can be calculated as:

10 $$\begin{aligned} & {\textbf{H}}^0\triangleq \frac{\underline{\text {mat} ( {\textbf{B}}{\textbf{p}} )}+\overline{\text {mat} ( {\textbf{B}}{\textbf{p}} )}}{2}, {\textbf{H}}^1\triangleq \frac{\underline{\varvec{\Theta }}+\overline{\varvec{\Theta }}}{2}, {\textbf{H}}^2\triangleq -\frac{ \underline{\text {mat} ( {\textbf{B}}{\textbf{p}} )} \underline{\varvec{\Theta }}+ \overline{\text {mat} ( {\textbf{B}}{\textbf{p}} )} \overline{\varvec{\Theta }} }{2} \end{aligned}$$

11 $$\begin{aligned} & {\textbf{g}}^0\triangleq \frac{\underline{(-2 {\textbf{A}} {\textbf{p}} )} + \overline{(-2 {\textbf{A}} {\textbf{p}})}}{2}, {\textbf{g}}^1\triangleq \frac{\underline{\varvec{\theta }}+\overline{\varvec{\theta }}}{2}, {\textbf{g}}^2\triangleq \frac{\underline{(-2 {\textbf{A}} {\textbf{p}})}\underline{\varvec{\theta }}+\overline{(-2 {\textbf{A}} {\textbf{p}}) }\overline{\varvec{\theta }}}{2} \end{aligned}$$

Here we use underline (resp. overline) to denote the lower (resp. upper) bounds of terms. It is worth noting that the range $[\underline{\varvec{\Theta }}$, $\overline{\varvec{\Theta }}]$ of $\varvec{\Theta }$ can be computed from the range $[\underline{\varvec{\theta }} ,\overline{\varvec{\theta }}]$ of $\varvec{\theta }$ based on the relation $\varvec{\Theta }=\varvec{\theta } \varvec{\theta }^\top$. This is explained in detail in the following section titled “[Sec Sec8].”

For the quadratic equality constraint, we can relax the constraint $\varvec{\Theta } = \varvec{\theta } \varvec{\theta }^\top$ into semidefinite constraint $\varvec{\Theta } \succeq \varvec{\theta } \varvec{\theta }^\top$.

Taking into account all the above considerations, we finally arrive at the following lower bound problem:

12a $$\begin{aligned} & \min _{ p, \varvec{\Theta }, \varvec{\theta }} E_{l}(\varvec{\Theta },\varvec{\theta },{\textbf{p}}){=} \text {trace}({\textbf{H}}^0\varvec{\Theta }) +\text {trace}({\textbf{H}}^1 \text {mat} ( {\textbf{B}}{\textbf{p}} ) ) +\text {trace}({\textbf{H}}^2 1 1^\top ) {+} \varvec{\theta }^\top {\textbf{g}}^0 + ({\textbf{g}}^1)^\top (-2{\textbf{A}}{\textbf{p}}) +1^\top {\textbf{g}}^2 {+} {\varvec{\rho }}^\top {\textbf{p}} \end{aligned}$$

12b $$\begin{aligned} & s.t.\ \begin{bmatrix} \varvec{\Theta } & \varvec{\theta } \\ \varvec{\theta }^\top & 1 \end{bmatrix} \succeq 0,\ {\textbf{p}}\in \Omega ,\ \ \underline{\varvec{\theta }}\le \varvec{\theta }\le \overline{\varvec{\theta }} \end{aligned}$$

Here we employ the fact that $\varvec{\Theta } \succeq \varvec{\theta } \varvec{\theta }^\top$ is equivalent to the constraint $$\begin{bmatrix} \varvec{\Theta } & \varvec{\theta } \\ \varvec{\theta }^\top & 1 \end{bmatrix} \succeq 0$$.

It is apparent that this problem can be decomposed into the following two subproblems only involving $(\varvec{\Theta },\varvec{\theta })$ or *p*:

13a $$\begin{aligned} \text {subproblem 1:}\quad&\min _{ \varvec{\Theta }, \varvec{\theta }} \quad \text {trace}({\textbf{H}}^0\varvec{\Theta }) + \varvec{\theta }^\top {\textbf{g}}^0 \end{aligned}$$

13b $$\begin{aligned}&s.t.\ \begin{bmatrix} \varvec{\Theta } & \varvec{\theta } \\ \varvec{\theta }^\top & 1 \end{bmatrix} \succeq 0,\ \underline{\varvec{\theta }}\le \varvec{\theta }\le \overline{\varvec{\theta }} \end{aligned}$$

and

14a $$\begin{aligned} \text {subproblem 2:} \quad&\min _{ p} \quad \text {trace}({\textbf{H}}^1 \text {mat} ( {\textbf{B}}{\textbf{p}} ) ) + ({\textbf{g}}^1)^\top (-2{\textbf{A}}{\textbf{p}}) + \varvec{{\rho }}^\top {\textbf{p}} \end{aligned}$$

14b $$\begin{aligned}&s.t.\ {\textbf{p}}\in \Omega \end{aligned}$$

Subproblem 1 is a low-dimensional semidefinite program which can be solved by solvers such as SDPNAL+^27^.

Subproblem 2 is an $n_p-$cardinality linear assignment problem which can be transformed into a standard linear assignment problem^28^ and then efficiently solved by combinatorial optimization algorithms such as LAPJV^29^.

### Branching strategy

Based on the result about bilinear convex envelope linearization as described previously, for the bilinear term $-2\varvec{\theta }^\top {\textbf{A}} {\textbf{p}}$, for its linear lower bound function to converge to it, we only need to branch the range of $\varvec{\theta }$, while keeping the range of $-2{\textbf{A}} {\textbf{p}}$ fixed. Here the range of $-2{\textbf{A}} {\textbf{p}}$ can be computed as

15 $$\begin{aligned} \min _{{\textbf{p}}\in \Omega } (-2{\textbf{A}} {\textbf{p}})_i \le (-2{\textbf{A}} {\textbf{p}})_i \le \max _{{\textbf{p}}\in \Omega } (-2{\textbf{A}} {\textbf{p}})_i \end{aligned}$$

Similarly, for the bilinear term $\text {trace}(\text {mat} ( {\textbf{B}}{\textbf{p}}) \varvec{\Theta })$, we only need to branch the range of $\varvec{\theta }$ (since the range of $\varvec{\theta }$ determines the range of $\varvec{\Theta }$ as shown in the sequel) while keeping the range of $\text {mat} ( {\textbf{B}}{\textbf{p}})$ fixed. Here the range of $\text {mat} ({\textbf{B}}{\textbf{p}} )$ can be computed as

16 $$\begin{aligned} \min _{{\textbf{p}}\in \Omega } [\text {mat} ( {\textbf{B}}{\textbf{p}} )]_{ij} {\le } [\text {mat} ( {\textbf{B}}{\textbf{p}} )]_{ij} {\le } \max _{{\textbf{p}}\in \Omega } [\text {mat} ( {\textbf{B}}{\textbf{p}} )]_{ij} \end{aligned}$$

We note that both (15) and (16) are $n_p-$cardinality linear assignment problems which can be efficiently solved by aforementioned algorithms.

In conclusion, in our BnB algorithm, we only need to branch over $\varvec{\theta }.$ Therefore, the dimension of the branching space of our BnB algorithm is low, enabling the proposed algorithm to converge quickly.

### The range of $\Theta$ from the range of $\theta$

Based on the relation $\varvec{\Theta }=\varvec{\theta } \varvec{\theta }^\top$, given the range $\left[ \underline{\varvec{\theta }}, \overline{\varvec{\theta }}\right]$ of $\varvec{\theta }$, we can compute the range $\left[ \underline{\varvec{\Theta }}, \overline{\varvec{\Theta }}\right]$ of $\varvec{\Theta }$ as:

$$\begin{aligned} {[}\underline{\varvec{\Theta }_{ij}},\overline{\varvec{\Theta }_{ij}}]= {\left\{ \begin{array}{ll} {[}\underline{\varvec{\theta }_i},\overline{\theta _i}] * [ \underline{\theta _j}, \overline{\theta _j}], & \text {if}\ i\ne j\\ {[}\underline{\theta _i},\overline{\theta _i}]^2, & \text {if}\ i= j \end{array}\right. } \end{aligned}$$

where, according to the interval arithmetic, the product operator is defined as

$$\begin{aligned} {[}\underline{\theta _i},\overline{\theta _i}] * [ \underline{\theta _j}, \overline{\theta _j}] = [\min (\underline{\theta _i}\underline{\theta _j}, \underline{\theta _i}\overline{\theta _j}, \overline{\theta _i}\underline{\theta _j}, \overline{\theta _i}\overline{\theta _j} ), \max (\underline{\theta _i}\underline{\theta _j}, \underline{\theta _i}\overline{\theta _j}, \overline{\theta _i}\underline{\theta _j}, \overline{\theta _i}\overline{\theta _j} ) ] \end{aligned}$$

and the square operator is defined as

$$\begin{aligned} {[}\underline{\theta _i},\overline{\theta _i}]^2 = {\left\{ \begin{array}{ll} {[} \min (\underline{\theta _i}^2, \overline{\theta _i}^2 ), \max (\underline{\theta _i}^2, \overline{\theta _i}^2 ) ], & \text {if}\ 0\notin [\underline{\theta _i}, \overline{\theta _i}] \\ {[} 0, \max (\underline{\theta _i}^2, \overline{\theta _i}^2 ) ], & \text {if}\ 0\in [\underline{\theta _i}, \overline{\theta _i}] \end{array}\right. } \end{aligned}$$

### Upper bound

By substituting the solutions for $\textbf{p}$ and $\varvec{\theta }$ obtained during the computation of the lower bound into $E(\textbf{p}, \varvec{\theta })$, we can get an upper bound. However, the $\varvec{\theta }$ solution may significantly deviate from the optimal $\varvec{\theta }$, especially in the early iterations of the BnB algorithm. This can result in a poor upper bound.

To tackle this issue, we can substitute the $\textbf{p}$ solution into $E(\textbf{p}) \triangleq \min _{\varvec{\theta }} E(\textbf{p}, \varvec{\theta })$, which also provides a valid upper bound. The analytic form of $E(\textbf{p})$ can be derived by recognizing that $E(\textbf{p}, \varvec{\theta })$ is a convex quadratic function of $\varvec{\theta }$, as indicated in Eq. (4a). Therefore, the optimal $\varvec{\theta }$ that minimizes $E(\textbf{p}, \varvec{\theta })$ can be found by solving the Eq. $\frac{\partial E}{\partial \varvec{\theta }} = 0$. The solution is:

17 $$\begin{aligned} \varvec{\hat{\theta }} = \text {mat}( \textbf{B} \textbf{p}) ^{-1} \textbf{A} \textbf{p} \end{aligned}$$

Here, the form of $E(\textbf{p}, \varvec{\theta })$ in Eq. (6) is utilized to solve for $\varvec{\theta }$. By substituting $\varvec{\hat{\theta }}$ back into $E(\textbf{p}, \varvec{\theta })$, we can eliminate $\varvec{\theta }$, thus obtaining the analytic form of $E({\textbf{p}})$ as:

18 $$\begin{aligned} E(\textbf{p}) \triangleq \min _{\varvec{\theta }} E(\textbf{p}, \varvec{\theta }) = -\textbf{p}^\top \textbf{A}^\top \text {mat}(\textbf{B} \textbf{p})^{-1} \textbf{A} \textbf{p} + \varvec{\rho }^\top \textbf{p} \end{aligned}$$

### Branch-and-bound

Based on the above preparations, we are now poised to apply the BnB algorithm to optimize $E({\textbf{p}},\varvec{\theta })$. we begin by utilizing the initial range of $\varvec{\theta }$ to establish the initial hypercube. In each iteration of the algorithm, we identify the hypercube with the lowest lower bound among all available hypercubes. This chosen hypercube is then subdivided to improve the global lower bound of the problem. Meanwhile, we update the upper bound by evaluating $E({\textbf{p}})$ using solutions obtained during the computation of the lower bounds. For a comprehensive overview, refer to Algorithm 1.

**Algorithm 1:**
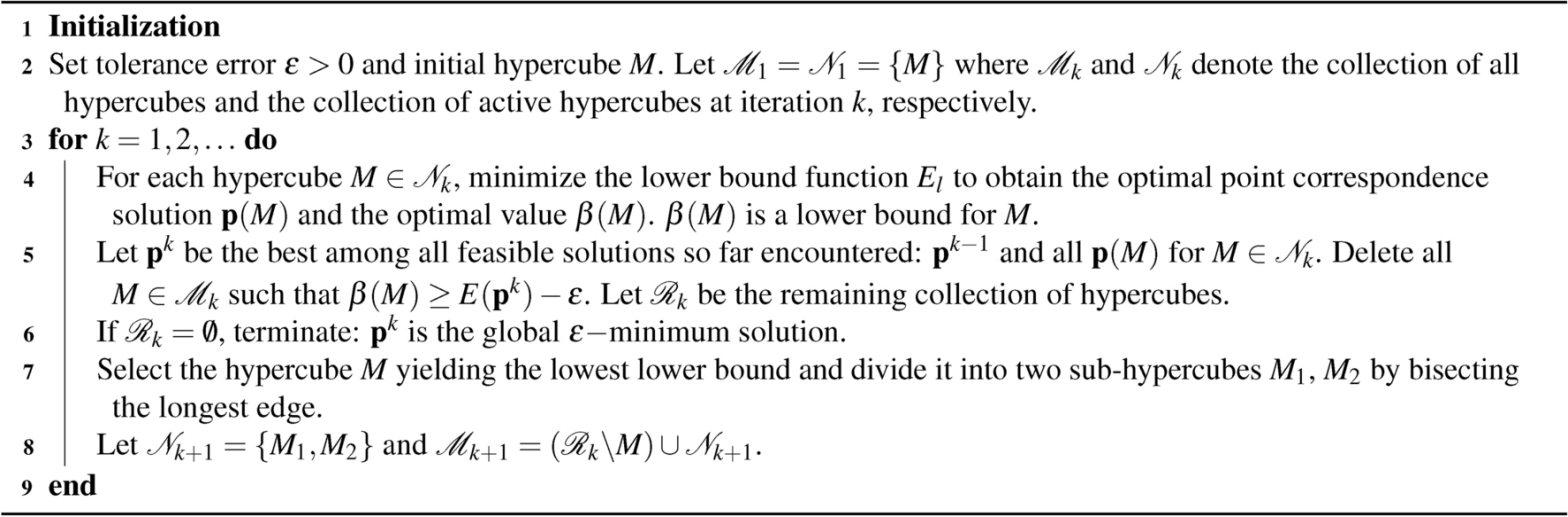
A BnB algorithm for minimizing *E*

### Convergence of the BnB algorithm

**Fig. 1:**
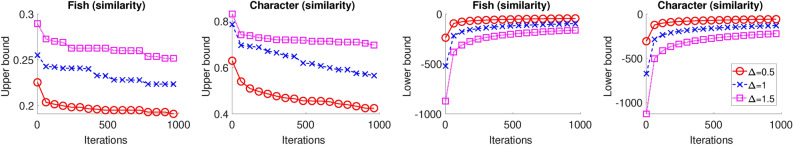
Upper (first row) and lower bounds (second row) generated in each iteration of our method. Our method is tested with the $n_p$ value chosen as the ground truth and with varying initial ranges of $\varvec{\theta }$: $[\varvec{\theta }_{gt}-\Delta , \varvec{\theta }_{gt}+ \Delta ]$, where $\varvec{\theta }_{gt}$ represents the ground truth $\varvec{\theta }$ solution. Here, the margin $\Delta$ takes values of 0.5, 1, and 1.5 respectively.

To assess the convergence of the proposed BnB algorithm, we use the separate outliers and inliers test described in the experimental section related to 2D registration, with an outlier-to-data ratio set at 0.3. The results are presented in Fig. 1.

Key observations include:

1. A narrower initial range of $\varvec{\theta }$ leads to a tighter duality gap, evidenced by improved upper and lower bounds. This suggests that leveraging additional information about the range of $\varvec{\theta }$ could effectively reduce the duality gap in practical applications.
2. Easier problem instances, such as the fish test compared to the character test, exhibit a tighter duality gap, resulting in faster convergence.

These observations suggest that using a fixed duality gap threshold as a stopping criterion for all problem types may not be ideal. It may happen that a threshold is set too small for a challenging problem, leading to long runtimes. Yet, the same threshold is set too large for an easier problem, causing premature termination. Therefore, in this work, we opt for maximum branching depth as our stopping criterion instead of a duality gap threshold.

While this practice means our method is no longer an $\epsilon$-globally optimal algorithm, it differs from traditional heuristic based methods by providing a measure of how far the solution is from the optimal one (i.e., the duality gap).

## Application 1: 2D similarity/affine registration

Our algorithm can handle 2D similarity and affine transformations since both of them can be written as the form of $T({\textbf{x}}|\varvec{\theta })=J({\textbf{x}})\varvec{\theta }$. A 2D similarity transformation has the form

$$\begin{aligned} T({\textbf{x}}|{\varvec{\theta }})=\begin{bmatrix} \theta _1 & -\theta _2 \\ \theta _2 & \theta _1 \end{bmatrix} \begin{bmatrix} x^1\\ x^2 \end{bmatrix} +\begin{bmatrix} \theta _3 \\ \theta _4 \end{bmatrix} =\begin{bmatrix}x^1& -x^2& 1& 0\\ x^2& x^1& 0& 1\end{bmatrix}\varvec{\theta } \end{aligned}$$

where $[\theta _3, \theta _4]^\top$ is translation and $\theta _1=s\cos (\phi )$ and $\theta _2=s\sin (\phi )$ with *s* being scale and $\phi$ being rotation angle.

A 2D affine transformation has the form

$$\begin{aligned} T({\textbf{x}}|{\varvec{\theta }})=\begin{bmatrix} \theta _1 & \theta _2 \\ \theta _3 & \theta _4 \end{bmatrix} \begin{bmatrix} x^1\\ x^2 \end{bmatrix} +\begin{bmatrix} \theta _5 \\ \theta _6 \end{bmatrix} =\begin{bmatrix} x^1& x^2& 0& 0& 1& 0\\ 0& 0& x^1& x^2& 0& 1 \end{bmatrix} \varvec{\theta } \end{aligned}$$

## Application 2: 3D rigid registration

Theoretically, our method can handle 3D affine transformation since it takes the form $T({\textbf{x}}|\varvec{\theta }) =J({\textbf{x}})\varvec{\theta }$. A 3D affine transformation has the form

$$\begin{aligned} T({\textbf{x}}|{\varvec{\theta }})=\begin{bmatrix} \theta _1 & \theta _2 & \theta _3 \\ \theta _4 & \theta _5 & \theta _6 \\ \theta _7 & \theta _8 & \theta _9 \end{bmatrix} \begin{bmatrix} x^1\\ x^2 \\ x^3 \end{bmatrix} +\begin{bmatrix} \theta _{10} \\ \theta _{11} \\ \theta _{12} \end{bmatrix} =\left[ \begin{array}{lccccccccccc} x^1& x^2& x^3& 0& 0& 0& 0& 0& 0& 1& 0& 0\\ 0& 0& 0& x^1& x^2& x^3& 0& 0& 0& 0& 1& 0\\ 0& 0& 0& 0& 0& 0& x^1& x^2& x^3& 0& 0& 1 \end{array}\right] \varvec{\theta } \end{aligned}$$

Nevertheless, a 3D affine transformation has many parameters, causing the resulting BnB algorithm to converge slowly. In contrast, 3D rigid transformation has much fewer parameters, Therefore, we aim to leverage the structure of 3D rigid transformation to improve efficiency of our method.

Using the angle-axis representation, a 3D rotation can be represented as a 3D vector ${\textbf{r}}$, with axis ${\textbf{r}}/\Vert {\textbf{r}}\Vert$ and angle $\Vert {\textbf{r}}\Vert$. The corresponding $3\times 3$ rotation matrix ${\textbf{R}}\in \mathbb{S}\mathbb{O}_3$ for ${\textbf{r}}$ can be obtained using matrix exponential map as^13^

19 $$\begin{aligned} {\textbf{R}}={\textbf{I}}_3+ \frac{[{\textbf{r}}]_\times \sin \Vert {\textbf{r}}\Vert }{\Vert {\textbf{r}}\Vert } +\frac{[{\textbf{r}}]_\times ^2(1-\cos \Vert {\textbf{r}}\Vert )}{\Vert {\textbf{r}}\Vert ^2} \end{aligned}$$

where $[\cdot ]_\times$ denotes the skew-symmetric matrix representation:

$$\begin{aligned} {[}{\textbf{r}}]_\times = \begin{bmatrix} 0 & -r_3& r_2\\ r_3& 0& -r_1 \\ -r_2 & r_1 & 0 \end{bmatrix} \end{aligned}$$

where $r_i$ is the *i*-th element of ${\textbf{r}}$.

A 3D rigid transformation is a special case of a 3D affine transformation, and since our method handles the more general affine case, it can be adapted to address 3D rigid transformations. The procedure is as follows:

We use the previously described algorithm for registration, where the transformation is modeled as a 3D affine transformation: $T(\textbf{x} \mid \textbf{R}, \textbf{t}) = \textbf{R} \textbf{x} + \textbf{t},$ with $\textbf{R}$ representing the linear component and $\textbf{t}$ the translation. However, the computed matrix $\textbf{R}$ may not necessarily be a valid rotation matrix. To correct this, we apply Eq. (19) and take the following steps to ensure that $\textbf{R}$ becomes a valid 3D rotation matrix:

(i) Instead of branching over $\textbf{R}$ and $\textbf{t}$, we branch over $\textbf{r}$ (the parameters defining the rotation) and $\textbf{t}$.
(ii) In each iteration of the branch-and-bound (BnB) algorithm, given the range of $\textbf{r}$, we compute the corresponding range for $\textbf{R}$ using Eq. (19). (The method for efficiently performing this computation will be discussed later.)

**Note**: In the above algorithm, we use Eq. (18) to compute the upper bound, which assumes an affine transformation. Although methods for computing the upper bound under rigid transformation constraints do exist (see Sect. 5.3 of Ref^25^), they typically result in higher upper bounds, which can slow down the convergence of our method. For this reason, we opt to use Eq. (18) for upper bound computation in this work.

### The range of ${\text{R}}$ from the range of ${\text{r}}$

Given a range $[\underline{{\textbf{r}}},\overline{{\textbf{r}}}]$ for ${\textbf{r}}$, based on (19), we can compute the range of ${\textbf{R}}$ as $\min \{ R_{ij}| \underline{{\textbf{r}}}\le {\textbf{r}}\le \overline{{\textbf{r}}}\} \le R_{ij} \le \max \{ R_{ij}| \underline{{\textbf{r}}}\le {\textbf{r}}\le \overline{{\textbf{r}}}\}$ via solvers such as the Matlab function *fmincon*. However, this process can be burdensome, particularly when executed iteratively as a subroutine of the BnB algorithm. In order to alleviate this computational burden, following^25^, we utilize precomputation techniques to enhance efficiency. Specifically, prior to initiating the BnB algorithm, we construct a regular grid, with a predefined width (set as 150 in this study), over the initial range $[\underline{{\textbf{r}}}_0,\overline{{\textbf{r}}}_0]$. Subsequently, we compute the corresponding $R_{ij}$ values for all grid points and store them. During the execution of the BnB algorithm, when presented with a range $[\underline{{\textbf{r}}},\overline{{\textbf{r}}}]$, we first identify the grid points falling within this range. Subsequently, we determine the minimum and maximum of the precomputed $R_{ij}$ values for these points, effectively approximating $\min \{ R_{ij}| \underline{{\textbf{r}}}\le {\textbf{r}}\le \overline{{\textbf{r}}}\}$ and $\max \{ R_{ij}| \underline{{\textbf{r}}}\le {\textbf{r}}\le \overline{{\textbf{r}}}\}$.

## Experiments

We implemented the proposed HBSP algorithm using Matlab R2023b. To assess its performance, we compared it with other methods on a computer equipped with 6-core CPUs running at 3.2 GHz.

For competing methods that generate point correspondences, we use these correspondences to compute the optimal affine transformation between two point clouds. We quantify the registration error as the root-mean-square distance between the transformed model inliers and their corresponding scene inliers.

Following^25^, we use maximum branching depth as the stopping criterion for our BnB algorithm, rather than tolerance error. Through empirical testing, we found that a maximum branching depth of 11 strikes a good balance between registration accuracy and computational efficiency.

**Fig. 2:**
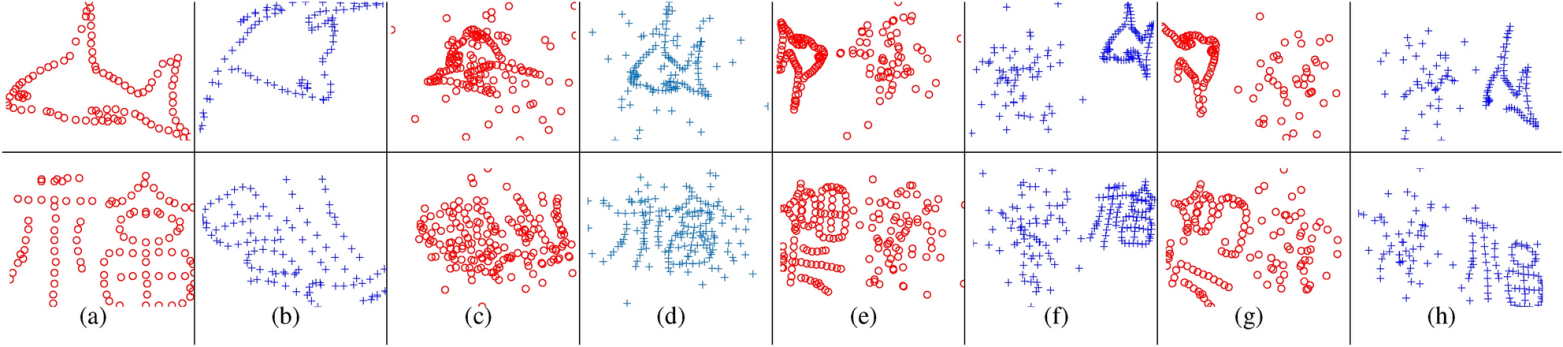
(**a**) and (**b**): Model point clouds and examples of scene point clouds from the deformation test. (**c**–**h**) Examples of model and scene point clouds for various tests: mixed outliers and inliers (**c**, **d**), separate outliers and inliers (**e**, **f**), and the occlusion+outlier test (**g**, **h**). In all cases, model points are marked with red circles, while scene points are represented by blue crosses.

**Fig. 3:**
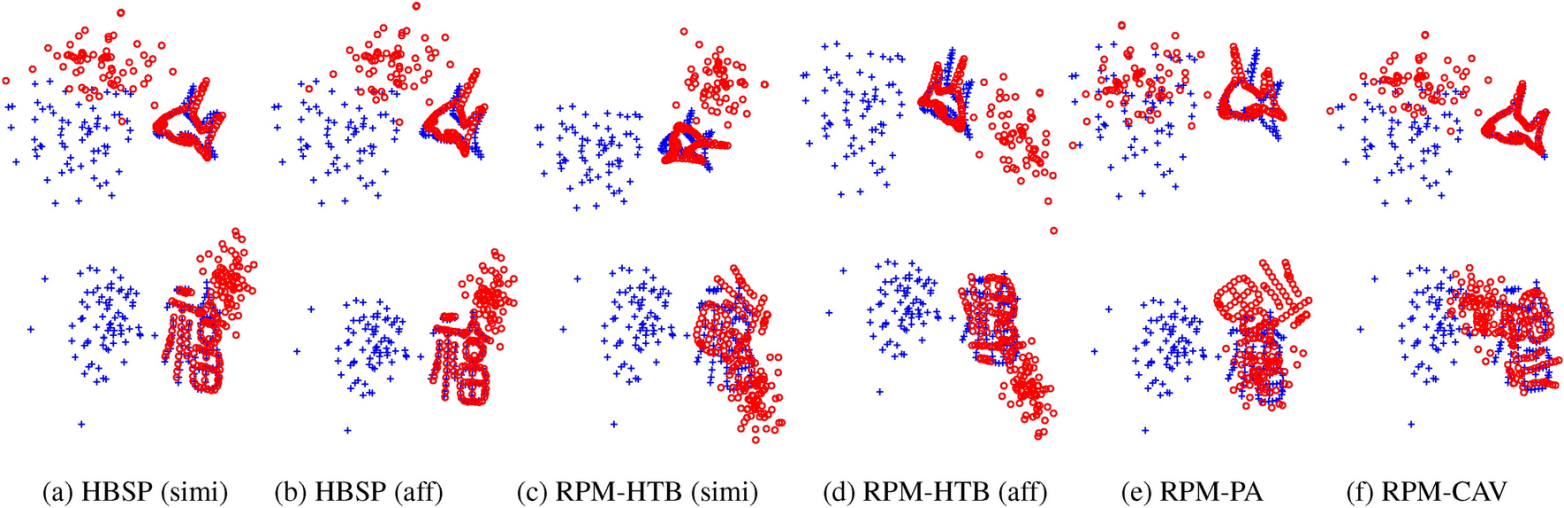
Registration results from various methods in the separate outliers and inliers test, using the ground truth value for $n_p$ across all methods.

**Fig. 4:**
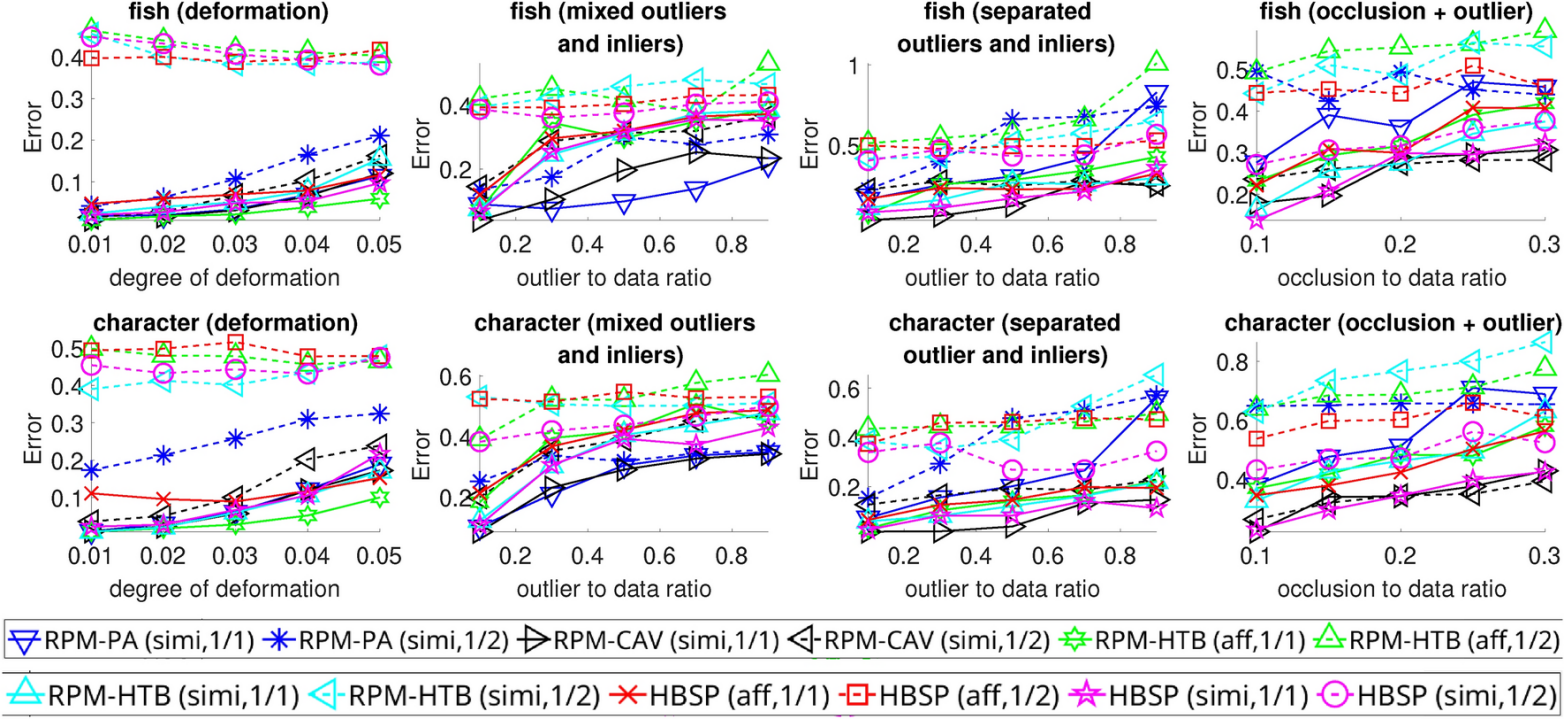
Average registration errors for HBSP, RPM-HTB, RPM-PA, and RPM-CAV across various $n_p$ values (ranging from $1/2$ to $1/1$ of the ground truth value) over 100 random trials. The tests included 2D deformation, mixed outliers and inliers, separate outliers and inliers, and occlusion+outlier scenarios.

### 2D registration

We compare our method against RPM-HTB^25^, RPM-PA^24^, and RPM-CAV^23^, all of which use global optimization techniques. These methods effectively handle partial overlap and allow for arbitrary similarity transformations between two point clouds, making them suitable candidates for comparison.

#### Synthetic data tests

 Synthetic data allows to evaluate specific aspects of an algorithm’s performance. We conduct four types of tests to assess robustness against various disturbances:

(i) Deformation test: The prototype shape undergoes non-rigid deformation to generate the scene point cloud.
(ii) Mixed outliers-inliers test: Random outliers are superimposed on the prototype shape to create both point clouds.
(iii) Separate outliers and inliers test: Random outliers are placed on different sides of the prototype shape to generate the two point clouds.
(iv) Occlusion+outlier test: The prototype shape is occluded to produce the two point clouds, followed by the addition of random outliers (with a fixed outlier-to-data ratio of 0.5) on different sides.

These tests are visually illustrated in Fig. 2. Additionally, to assess the method’s capability to handle arbitrary rotation and scaling, we apply random rotations and scalings within the range of $[0.5, 1.5]$ when generating the two point clouds. Figure 3 provides examples of registrations performed by different methods.

#### Results

 The registration errors produced by various methods are shown in Fig. 4. The results indicate that HBSP, with $n_p$ set to the ground truth, is robust against deformation and outliers when outliers are separate from the inliers. However, its robustness weakens when outliers are mixed with inliers.

In comparison, RPM-PA performs well when outliers are mixed with inliers but struggles when they are separate. RPM-CAV demonstrates robustness to all types of disturbances. RPM-HTB performs slightly worse than HBSP in tests involving outliers.

In terms of transformation options, HBSP exhibits greater robustness to disturbances when using similarity transformations compared to affine transformations.

The choice of $n_p$ also significantly affects HBSP’s performance. Specifically, using an $n_p$ value that deviates from the ground truth (e.g., when halved) yields poorer results. Nevertheless, HBSP is less sensitive to variations in the $n_p$ value than RPM-HTB, particularly in tests involving outliers and occlusion.

The average run times (in seconds) for the various methods are as follows: HBSP (affine): 136.0, HBSP (similarity): 122.8, RPM-PA: 9.8, RPM-CAV: 3.4, RPM-HTB (affine): 15.9, RPM-HTB (similarity): 10.6. The longer run times for our method can be attributed to the need to solve a semidefinite program in each iteration of the BnB algorithm.

### 3D registration

**Fig. 5:**
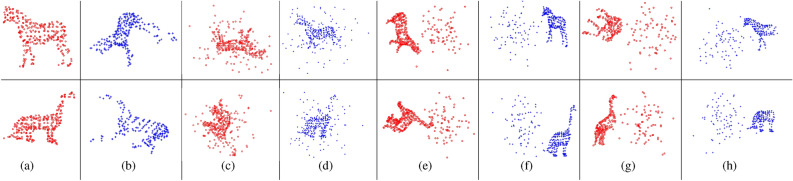
(**a**) and (**b**): Model point clouds and examples of scene point clouds from the deformation test. (**c**)–(**i**): Examples of model and scene point clouds for the mixed outliers and inliers test (**d**, **e**), the separate outliers and inliers test (**f**, **g**), and the occlusion+outlier test (**h**, **i**). In all cases, model points are shown as red circles, while scene points are represented by blue crosses.

**Fig. 6:**
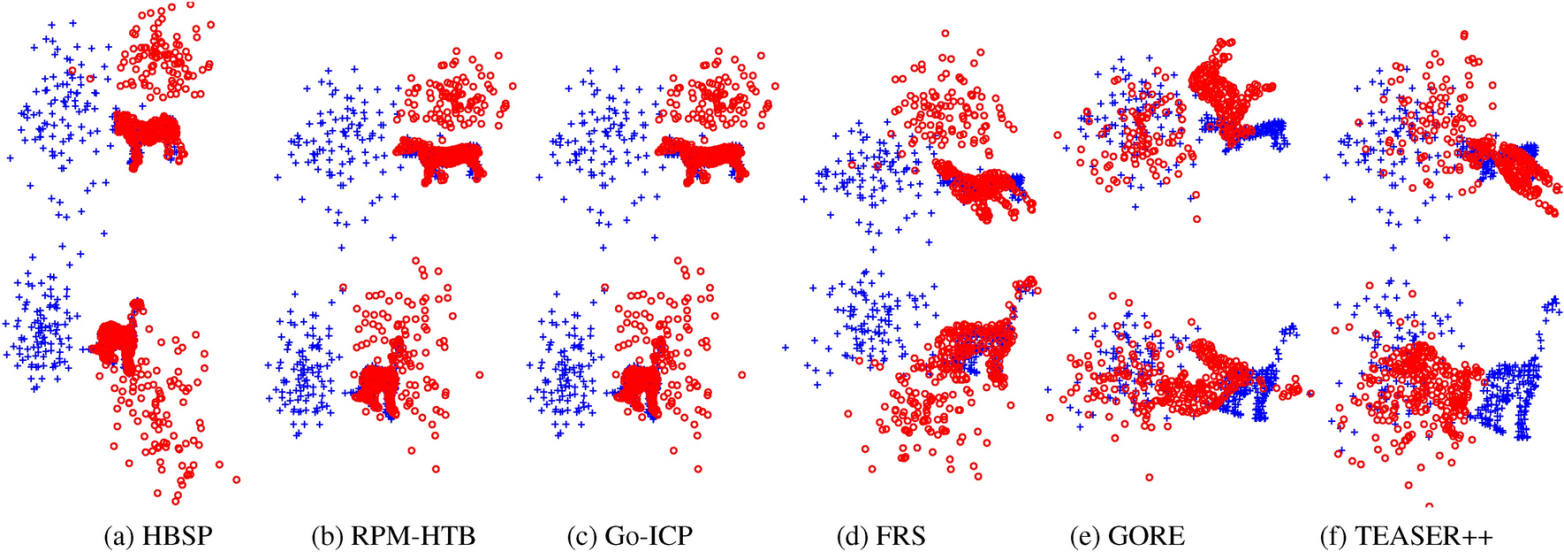
Examples of registration results from different methods in the separate outliers and inliers test, using the ground truth $n_p$ values for both RPM-HTB and Go-ICP.

**Fig. 7:**
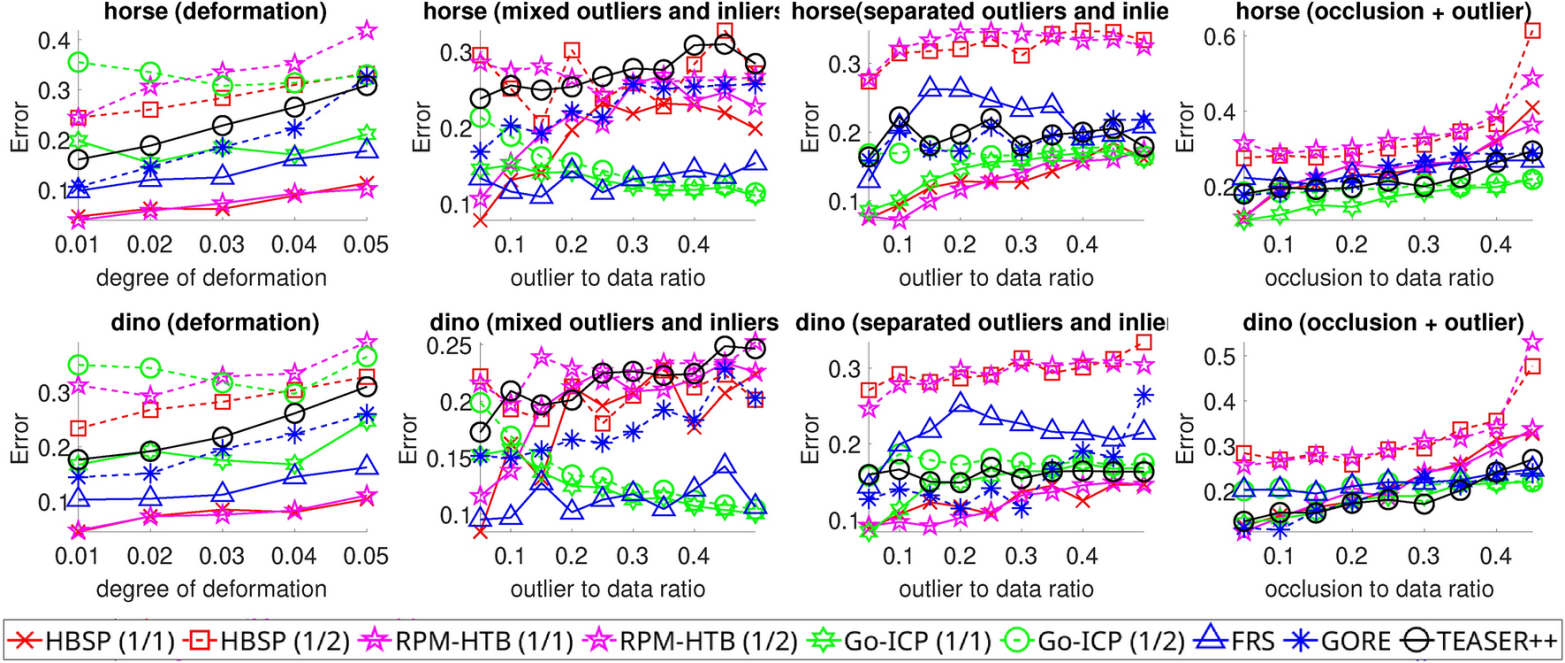
Average registration errors for HBSP, RPM-HTB, Go-ICP, FRS, GORE, and TEASER++ under varying $n_p$ values (ranging from $1/2$ to $1/1$ of the ground truth value) across 100 random trials. The tests included 3D deformation, mixed outliers and inliers, separate outliers and inliers, and occlusion+outlier scenarios.

Since RPM-PA and RPM-CAV are not efficient for the 3D case, they are not tested here. Instead, we compare our method with Go-ICP^13^, FRS^15^, GORE^30^ and TEASER++^19^. These methods are based on global optimization and allows arbitrary rigid transformation between two point clouds, making them suitable for comparison. For GORE and TEASER++, the FPFH feature descriptor is used to extract putative matches and the number of putative matches is set to 500.

#### Synthetic data tests

 Similar to the previous section, we conducted four types of tests to assess the methods’ robustness against various types of disturbances: (i) Deformation test. (ii) Mixed outliers and inliers test. (iii) Separate outliers and inliers test. (iv) Occlusion+outlier test. Refer to Fig. 5 for an illustration, and see Fig. 6 for examples of registrations performed by different methods.

#### Results

 Figure 7 displays the registration errors from various methods. The results indicate that HBSP, with $n_p$ set to the ground truth, is robust to deformation and separated outliers and inliers. However, its effectiveness decreases when outliers are mixed with inliers.

In contrast, GORE and TEASER++ struggle with deformation and outliers, especially when outliers are mixed with inliers, likely due to their reliance on feature descriptors. FRS is not robust when outliers are separate from inliers, and Go-ICP fails to handle non-rigid deformation effectively. RPM-HTB performs comparably to HBSP when $n_p$ is set to the ground truth but slightly worse when $n_p$ deviates. In terms of parametric robustness, HBSP performs significantly better when $n_p$ closely matches the ground truth.

The average run times (in seconds) for the various methods are as follows: HBSP: 205.9, RPM-HTB: 25.9, Go-ICP: 0.5, FRS: 235.2, GORE: 92.3, TEASER++: 3.3.

## Conclusion

In this work, we introduced a BnB-based point cloud registration algorithm that effectively aligns partially overlapping point clouds while remaining invariant to the corresponding transformation. This method offers several advantages: it computes the lower bound using an efficient linear assignment algorithm and low-dimensional semidefinite programming. Additionally, the dimensionality of the branching space matches the number of transformation parameters, leading to good convergence.

Overall, the experimental results indicate that our method is well-suited for scenarios involving significant deformations, where outliers can be clearly separated from inliers, and when the number of matches closely aligns with the ground truth.

One drawback of our method is its relatively high runtime. In the future, we aim to enhance its efficiency further.

## Data Availability

Data are available from the corresponding author on reasonable request.
